# Hypernatremia secondary to partial urinary tract obstruction: a case report

**DOI:** 10.3389/fendo.2026.1738471

**Published:** 2026-03-23

**Authors:** Shizhe Zhou, Tianyuan Li, Chenxiang Cao, Jianzhong Xiao, Zhaoxiang Liu

**Affiliations:** Department of Endocrinology and Metabolism, Beijing Tsinghua Changgung Hospital, School of Clinical Medicine, Tsinghua University, Beijing, China

**Keywords:** hyperglycemic hyperosmolar state, hypernatremia, prostatic hyperplasia, type 2 diabetes, urinary tract obstruction

## Abstract

**Introduction:**

This case report describes a 52-year-old male with a 6-month history of polydipsia and polyuria, and increased urinary foam for 2 months. He was initially diagnosed with type 2 diabetes mellitus complicated by hyperglycemic hyperosmolar state (HHS). Despite stabilization of blood glucose, he developed refractory, unexplained severe hypernatremia.

**Methods:**

The patient received standard care including insulin therapy and fluid resuscitation. Due to the persistent hypernatremia and the known concurrent conditions of benign prostatic hyperplasia, bilateral hydronephrosis with ureteral dilation, and bladder calculi, he subsequently underwent transurethral holmium laser enucleation of the prostate and cystolithotripsy.

**Results:**

Following the urological surgery, the patient's serum sodium level rapidly normalized, decreasing from a peak of 162.3 mmol/L to 139.7 mmol/L, with complete resolution of the hypernatremia.

**Discussion:**

This case illustrates that partial urinary tract obstruction can induce hypernatremia by impairing renal medullary concentrating ability and sodium-water reabsorption, leading to a disproportionate loss of water relative to sodium. It highlights the critical importance of actively investigating urinary tract obstruction in patients with unexplained hypernatremia, particularly those with urological symptoms or elderly males. Early surgical intervention to relieve the obstruction can significantly improve the prognosis.

## Introduction

Hypernatremia is a common electrolyte disturbance in clinical practice, with diverse etiologies requiring comprehensive evaluation incorporating patient history, physical examination, and ancillary investigations. As a rare cause of hypernatremia, urinary tract obstruction (UTO) has been documented in only three case reports over the past four decades, rendering it easily overlooked. Peterson et al. reported a case of a 78-year-old male with bladder outlet obstruction due to prostatic hyperplasia, presenting with serum sodium as high as 188 mmol/L. Waise and Fisken reported two elderly male patients (aged 79 and 82 years, respectively) with serum sodium levels of 159 mmol/L and 156 mmol/L, both of whom achieved normalization of serum sodium following relief of obstruction (1, 2). The prevalence of hypernatremia among hospitalized patients ranges from 1% to 3%, with higher incidence rates observed in intensive care units (3, 4). Most cases result from water loss (either renal or extrarenal) or increased sodium intake, while UTO remains a rare and frequently unrecognized cause of persistent hypernatremia. Literature searches reveal only a limited number of case reports documenting hypernatremia secondary to partial urinary tract obstruction, with merely three cases involving elderly males published over the past forty years. This paucity of published cases may reflect that this electrolyte disturbance typically resolves spontaneously following treatment of the underlying obstruction, and thus has not received adequate clinical or research attention. Through this case of hypernatremia secondary to partial urinary tract obstruction, we explore the pathophysiological mechanisms, clinical characteristics, and therapeutic strategies, aiming to enhance clinical awareness of this condition and emphasize the importance of considering partial urinary tract obstruction in the differential diagnosis of unexplained persistent hypernatremia, particularly in middle-aged and elderly male patients with voiding dysfunction.

## Case presentation

### Patient information

A 52-year-old male patient was admitted on April 29, 2025, with chief complaints of polydipsia and polyuria for 6 months and increased urinary foam for 2 months.

### Clinical presentation

Six months prior to admission, the patient developed polydipsia and polyuria without apparent precipitating factors, experiencing nocturia 3–4 times per night, accompanied by weight loss (from 88 kg to 82 kg). He denied urinary foam or visual disturbances. At that time, glycated hemoglobin (HbA1c) was 7.5% (reference: 4.27-6.07%) and fasting blood glucose (FBG) was 5.9 mmol/L (reference: 3.9-6.1 mmol/L); however, these findings were not addressed. Two months before admission, the patient experienced fatigue with occasional increased urinary foam, and his weight further decreased to 70 kg, without seeking medical attention. One day prior to admission, outpatient laboratory tests revealed FBG of 24.96 mmol/L (reference: 3.9-6.1 mmol/L), HbA1c of 12.5% (reference: 4.27-6.07%), serum osmolality of 346 mOsm/kg H_2_O (reference: 275–295 mOsm/kg H_2_O), and urine ketones 1+ (reference: negative). The patient reported no nausea, vomiting, or altered consciousness. Past medical history was significant for hyperuricemia, hypercholesterolemia, hypertriglyceridemia, and benign prostatic hyperplasia. He denied alcohol consumption, had a 35-year smoking history (10 cigarettes per day), and had quit smoking 2 months prior. Family history was negative for diabetes mellitus.

Physical examination on admission: Temperature 36.5 °C, heart rate 74 beats/min, respiratory rate 17 breaths/min, blood pressure 134/86 mmHg. BMI 20.23 kg/m², waist circumference 87 cm. Cardiopulmonary and abdominal examinations, lower extremity examination, and diabetic foot screening (including dorsalis pedis pulse, pain and temperature sensation, vibration sense, and 10-g monofilament test) revealed no significant abnormalities.

### Diagnostic evaluation

Relevant ancillary investigations are summarized in **Table 1**. The patient presented with severe hyperglycemia (FBG 24.96 mmol/L), elevated plasma osmolality (≥320 mOsm/kg H_2_O), arterial blood gas analysis showing pH >7.30 and HCO_3_^-^ >15 mmol/L, weakly positive urine ketones, and elevated serum sodium, consistent with a diagnosis of hyperglycemic hyperosmolar state.

**Table 1:** Relevant ancillary investigations performed after admission.

Parameter	Before treatment	After treatment
Liver and Renal Function		
ALT (U/L)	27.5	
AST (U/L)	17.6	
Creatinine (μmol/L)	136	102
eGFR (ml/min/1.73m²)	51.177	72.464
Uric Acid (μmol/L)	494	
K (mmol/L)	4.28	3.1
Na (mmol/L)	162.3	153.5
Complete Blood Count		
WBC (× 109/L)	6.85	
HGB (g/L)	130	
PLT (× 10/L)	224	
Arterial Blood Gas		
pH	7.43	7.35
PCO2 (mmHg)	32	40
PO2 (mmHg)	112	96
HCO3- (mmol/L)	21.2	22.1
Na (mmol/L)	156	154
K (mmol/L)	4.1	3.7
Cl (mmol/L)	124	121
Glu (mmol/L)	26.6	18.0
Lac (mmol/L)	1.3	0.7
AG (mmol/L)	15	15
Tumor Markers		
T-PSA (ng/mL)	7.508	
F-PSA (ng/mL)	2.478	
CA19-9 (U/mL)	53.32	
SCC-Ag (ng/ml)	1.0	
CA15-3 (U/mL)	10.1	
CEA (ng/mL)	6.27	
AFP (ng/mL)	3.57	
CYFRA21-1 (ng/ml)	6.07	

### Treatment intervention

Initial management consisted of fluid resuscitation, continuous intravenous insulin infusion, and correction of electrolyte abnormalities (0.9% normal saline 2500 mL, lactated Ringer’s solution 500 mL; subsequently changed to 5% glucose-saline 500 mL with insulin; intravenous potassium supplementation; oral fluid intake 2500 mL; total fluid volume: 6000 mL). Follow-up arterial blood gas analysis revealed: pH 7.35 (reference: 7.34-7.44), PCO_2_ 40 mmHg (reference: 35–45 mmHg), PO_2_ 96 mmHg (reference: 75–100 mmHg), HCO_3_^-^ 22.1 mmol/L (reference: 22–26 mmol/L), Na^+^ 154 mmol/L (reference: 135–155 mmol/L), K^+^ 3.7 mmol/L (reference: 3.5-5.5 mmol/L), Cl^-^ 121 mmol/L (reference: 98–107 mmol/L), glucose 18.0 mmol/L (reference: 3.3-6.1 mmol/L), lactate 0.7 mmol/L (reference: 0.4-2.2 mmol/L), anion gap 15 mmol/L (reference: 8–16 mmol/L).

The regimen was subsequently transitioned to insulin glargine and insulin aspart for glycemic control. After blood glucose stabilization, a steamed bread meal test was performed, which demonstrated delayed insulin secretion without absolute deficiency and negative islet autoantibodies, consistent with type 2 diabetes mellitus. The hypoglycemic regimen was changed to sitagliptin-metformin 1 tablet twice daily and gliclazide sustained-release 30 mg once daily (before breakfast), with monitored fasting glucose of 7.2 mmol/L.

**Figure 1:**
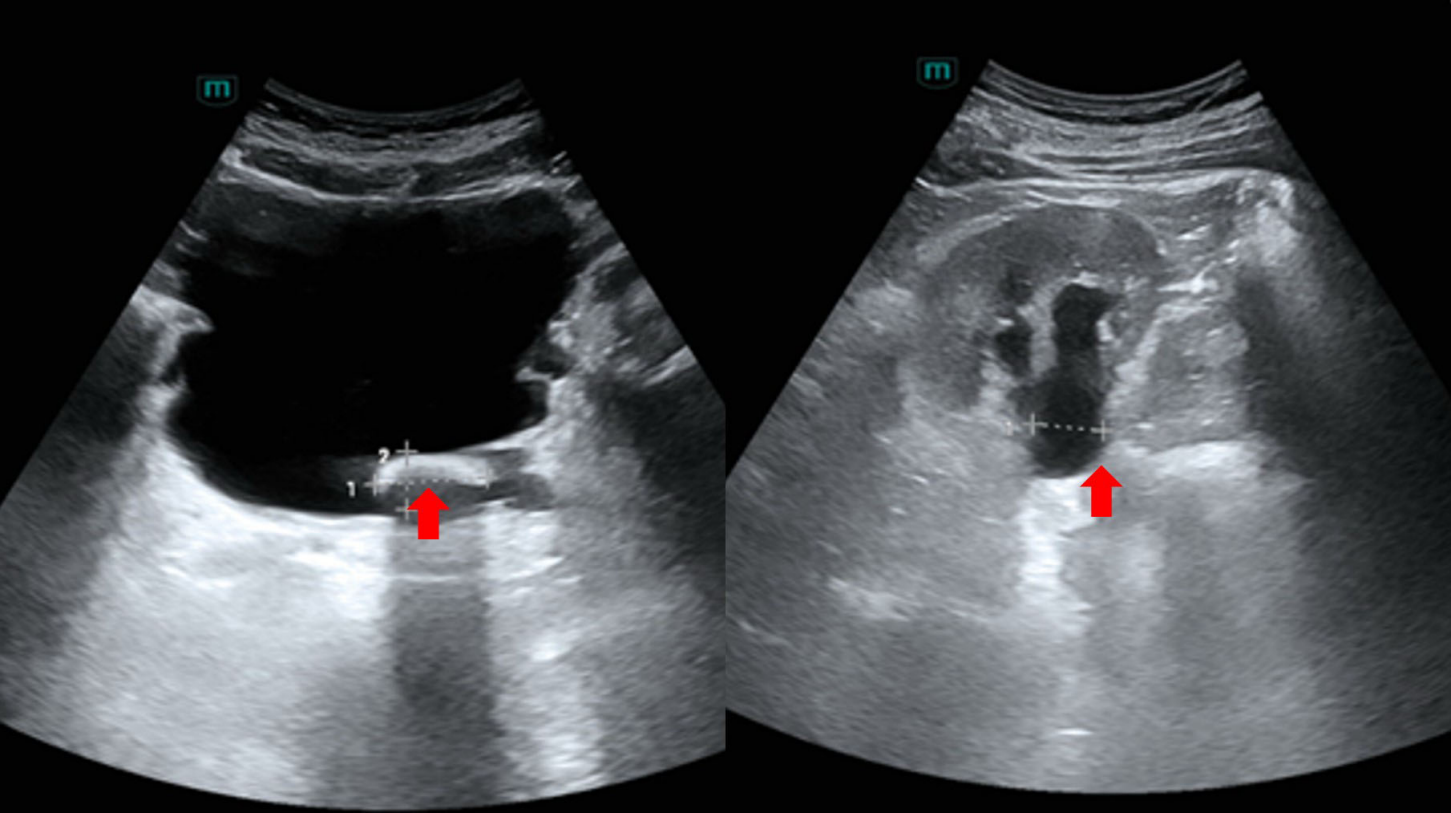
Urological ultrasonography. Both kidneys demonstrated normal size and morphology with clear capsules and homogeneous parenchymal echogenicity. The intrarenal architecture was clearly visualized. Bilateral pelvic separation was observed, measuring approximately 1.7 cm on the right and 1.8 cm on the left, without obvious space-occupying lesions. Bilateral ureteral dilation was noted, measuring approximately 0.9 cm on the right and 0.5 cm on the left. The bladder was well-distended with thickened, irregular walls showing trabeculated changes consistent with detrusor hypertrophy. An intraluminal hyperechoic mass measuring approximately 2.8 cm × 1.4 cm was identified, with posterior acoustic shadowing. The prostate was enlarged, measuring approximately 4.9 cm × 5.6 cm × 4.3 cm, with heterogeneous internal echogenicity and hyperechoic foci at the interface between the inner and outer glands.

**Figure 2:**
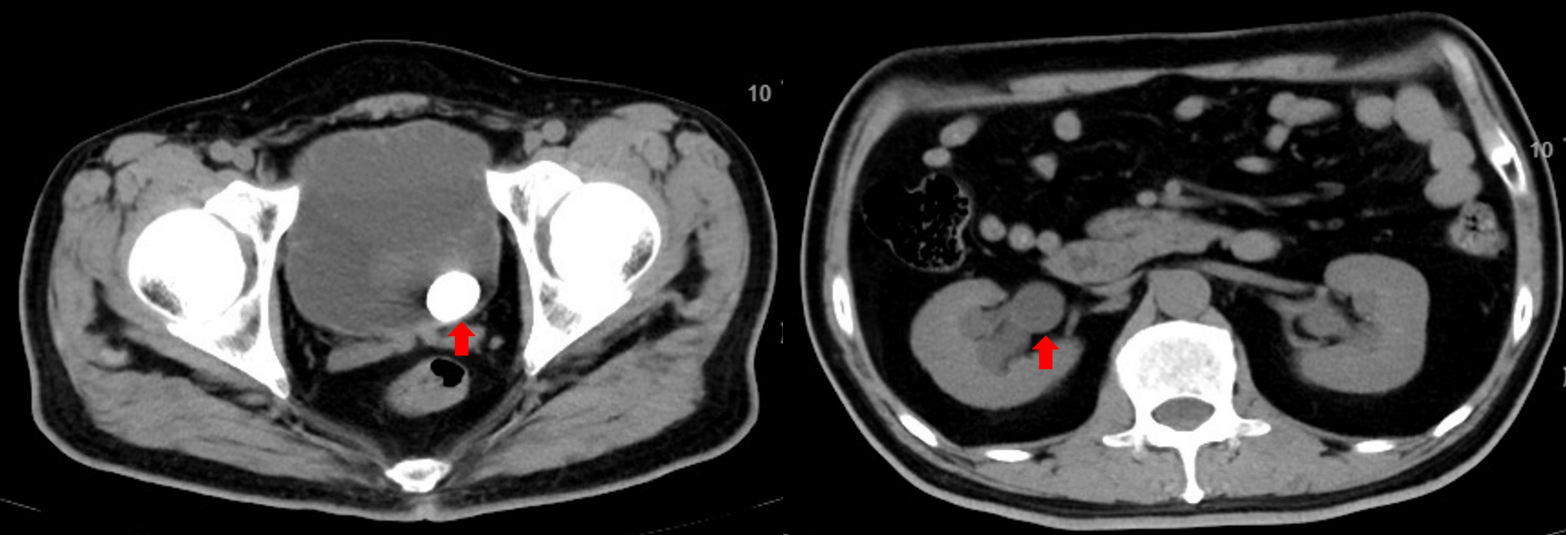
Abdominal computed tomography. Both kidneys were normal in position and size with regular morphology. Bilateral renal parenchyma demonstrated round hypodense lesions, the largest measuring approximately 9 mm in diameter. Bilateral pelvicalyceal systems and ureters were dilated with fluid accumulation. Bilateral perirenal fat planes were clear. The bladder was overdistended, containing a nodular hyperdense lesion measuring approximately 23 mm × 18 mm; a small diverticulum was noted on the right lateral wall. The prostate was enlarged with a diameter of approximately 54 mm, demonstrating punctate hyperdense foci and protruding into the bladder. Bilateral seminal vesicles were symmetrical with homogeneous density.

The patient’s serum sodium fluctuated between 153.4–154 mmol/L. He reported troublesome urinary frequency and nocturia and had consciously reduced fluid intake, without obvious symptoms of dehydration such as thirst or dry skin. Tumor markers showed no significant abnormalities. Urological ultrasonography revealed bilateral hydronephrosis, bilateral ureteral dilation, bladder calculus, trabeculated bladder indicative of detrusor hypertrophy, and prostatic hyperplasia with calcifications (**Figure 1**). Abdominal CT demonstrated bladder calculus, bilateral pelvicalyceal and ureteral dilation with fluid accumulation, considered to be related to bladder overdistension (**Figure 2**). Urology consultation recommended: indwelling catheter placement, initiation of tamsulosin (0.2 mg once daily) and finasteride (5 mg once daily). Post-void residual volume was 364 mL; uroflowmetry revealed free flow rate with peak flow of 5 mL/s and voided volume of 74 mL. The patient was transferred to the urology department for transurethral holmium laser enucleation of the prostate and transurethral cystolithotripsy.

### Follow-up and outcomes

Within 24 hours following surgical intervention, serum sodium normalized to 139.7 mmol/L without additional fluid therapy beyond maintenance crystalloid infusion. At 4-month follow-up, serum sodium remained within normal limits at 140.8 mmol/L.

## Discussion

Hyperglycemic hyperosmolar state (HHS) is a severe acute complication of diabetes mellitus characterized by marked hyperglycemia, hyperosmolality, and dehydration without significant ketosis, commonly occurring in patients with type 2 diabetes. Insulin deficiency combined with elevated counter-regulatory hormones (glucagon, catecholamines, cortisol, and growth hormone) collectively contribute to this metabolic derangement (5–7), resulting in glycosuria and osmotic diuresis that ultimately leads to hypovolemia, progressive decline in glomerular filtration rate, severe hyperglycemia, and hypernatremia (8). Following aggressive fluid resuscitation and insulin-mediated glucose reduction, osmolality decreases, hypovolemia is corrected, glomerular filtration rate recovers, and hypernatremia typically resolves accordingly (9). In the present case, hypernatremia persisted despite adequate glycemic control, necessitating further investigation for other underlying etiologies.

**Figure 3:**
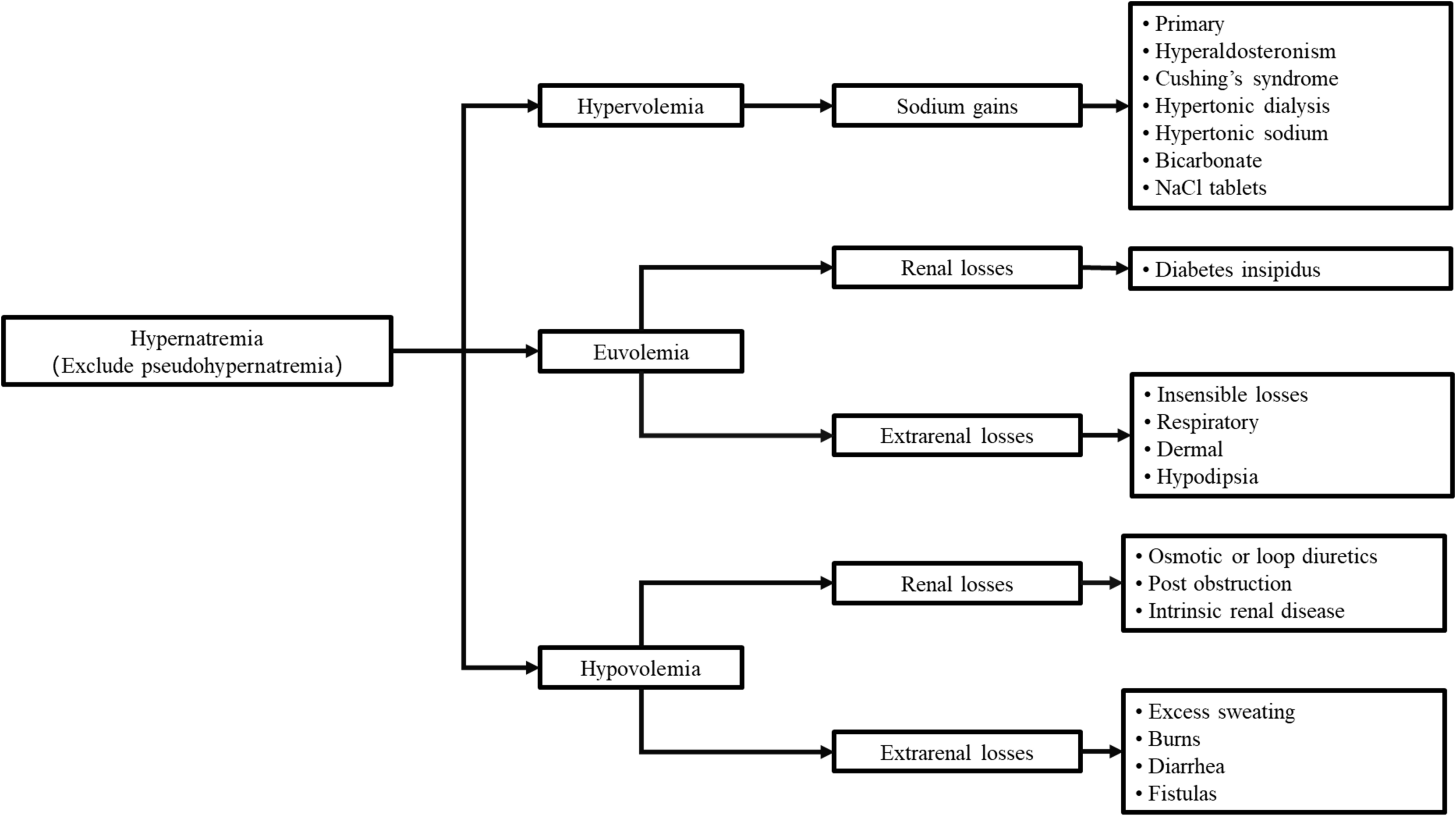
Causes of hypernatremia.

Hypernatremia (Na^+^ >145 mmol/L) is an electrolyte disturbance typically resulting from free water losses exceeding sodium losses (10, 11). This imbalance may arise from (1) net water loss, either as pure water (without sodium deficit) or hypotonic fluid (with sodium deficit), or (2) hypertonic sodium gain (12). Most cases of hypernatremia result from net water loss through renal or extrarenal pathways (coma or gastrointestinal losses) (13). Renal causes include central or nephrogenic diabetes insipidus, hyperglycemia-induced osmotic diuresis, post-obstructive diuresis, or mannitol administration (14–17). Hypertonic sodium gain typically results from clinical interventions or inadvertent sodium loading (14). The etiological classification of hypernatremia summarized by Giae Yun et al. (10) is presented in **Figure 3**.

After excluding pseudohypernatremia, this patient had no history or symptoms of extrarenal sodium loss, denied precipitating factors such as hypertonic sodium intake or excessive sodium supplementation, had not received diuretic therapy, denied history of hypertension, and blood pressure monitoring during hospitalization ranged from 125-138/78–88 mmHg without antihypertensive therapy. He denied history of adrenal adenoma, and cortisol levels were unremarkable (ACTH [8 AM] 20.1 ng/L, cortisol [8 AM] 385 nmol/L). RAAS evaluation (Ang II 65.60 pg/mL, aldosterone 61.31 pg/mL, renin 1.00 pg/mL, ARR 61.31) revealed low renin levels with suppressed aldosterone, consistent with volume expansion secondary to hypernatremia rather than primary aldosteronism. These findings effectively excluded mineralocorticoid excess as the cause of hypernatremia.

**Figure 4:**
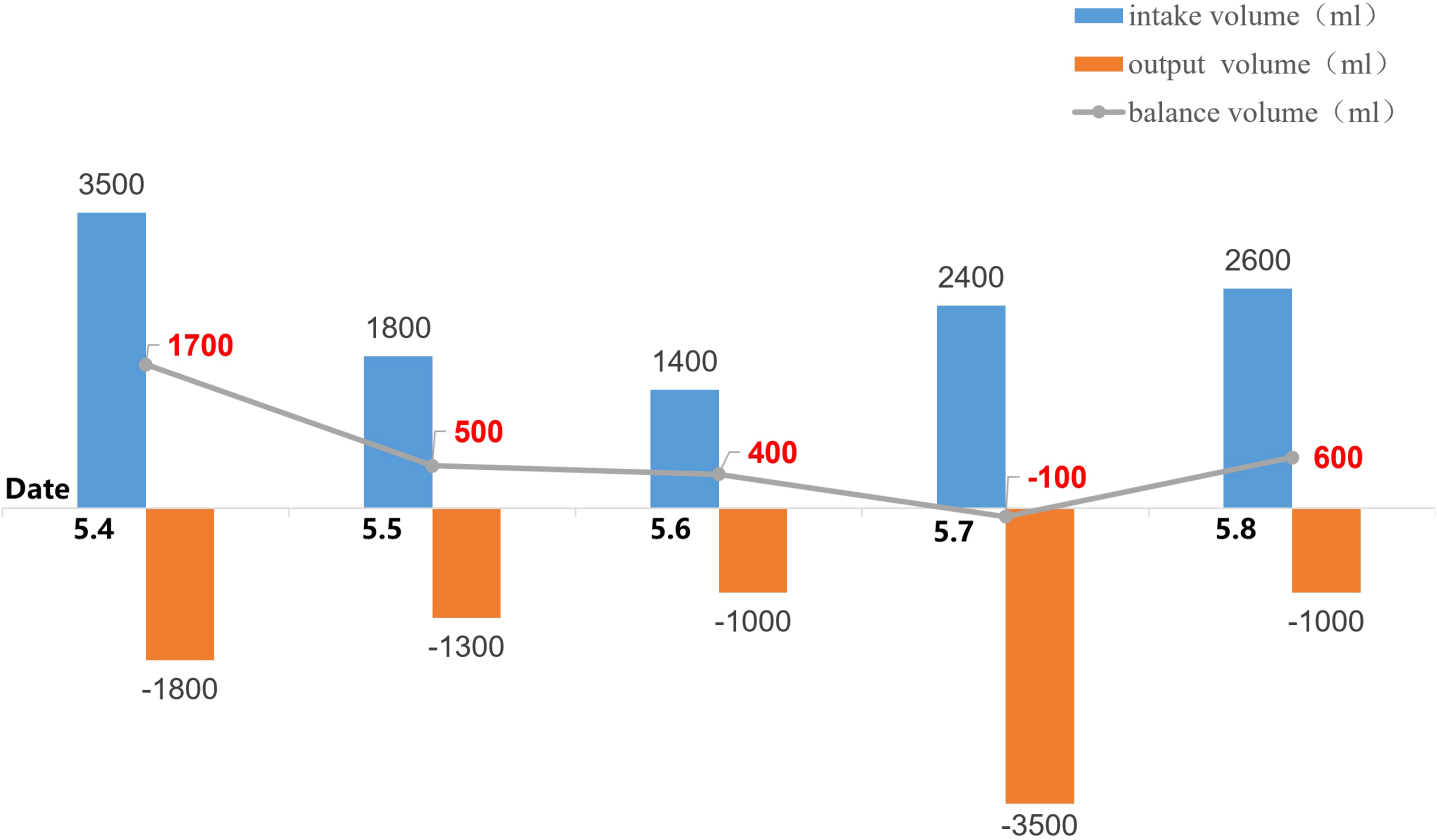
Input and output volume.

**Figure 5:**
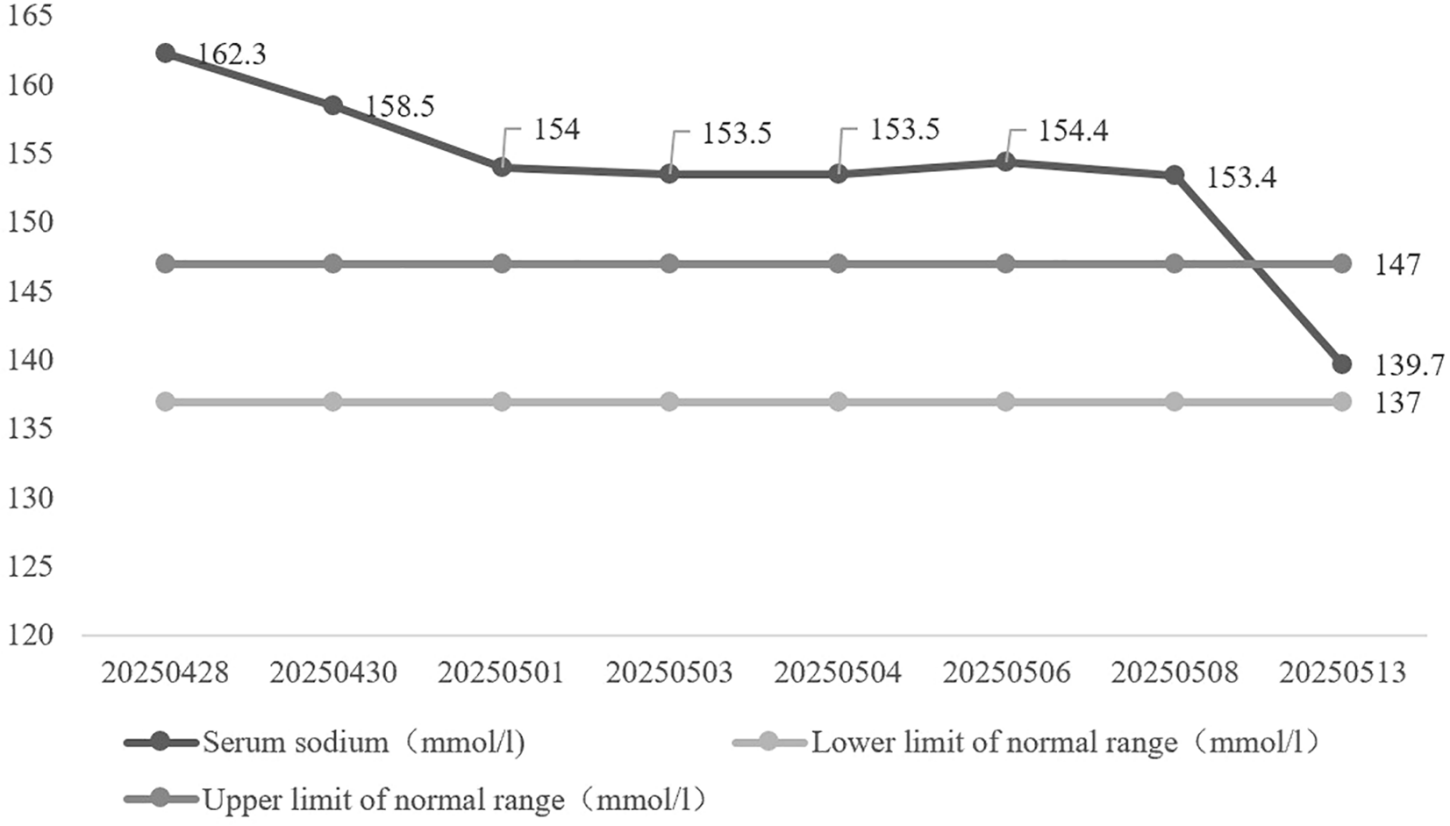
Change in blood sodium level.

Despite uncorrected hypernatremia, intake/output monitoring (**Figure 4**) revealed intake of 2000–2500 mL/day and output of 1800–2300 mL/day, without the massive polyuria (>3 L/day) expected in diabetes insipidus. Urine osmolality under non-fasting conditions (mOsm/kg H_2_O: 346→311) remained consistently >300 mOsm/kg H_2_O; specific gravity was 1.006, without obvious symptoms of dehydration such as thirst or dry skin, preliminarily excluding diabetes insipidus. Although elevated PSA was consistent with benign prostatic hyperplasia (confirmed by imaging), the mild elevations in CA19-9, CEA, and CYFRA21–1 were considered non-specific, likely attributable to acute metabolic stress, dehydration, and renal impairment rather than occult malignancy. No clinical or imaging evidence of malignancy was identified, and these values were expected to normalize following metabolic stabilization and relief of obstruction; concurrent malignancy was not considered. Follow-up eGFR was 72.464, and diabetic nephropathy-induced hypernatremia was not considered. Based on imaging findings and normalization of serum sodium following surgery (Na 139.7 mmol/L), with eGFR recovering to 89.066, hypernatremia secondary to partial urinary tract obstruction was diagnosed. At 4-month follow-up, serum sodium remained normal at 140.8 mmol/L (**Figure 5**).

Previous literature has documented three elderly male cases with hypernatremia secondary to partial urinary tract obstruction. All three patients had bladder outlet obstruction with hydronephrosis, with serum sodium levels of 188 mmol/L, 159 mmol/L, and 156 mmol/L, respectively. Hypernatremia resolved following relief of obstruction (1, 2).

Partial obstructive nephropathy impairs tubular function (particularly concentrating capacity), causing relative water loss—a mechanism analogous to the “concentrating hypernatremia” observed in diabetic HHS. Partial obstruction leads to elevated intrapelvic pressure, renal ischemia, and inflammatory responses, ultimately preventing maintenance of the normal hypertonic environment; once the medullary osmotic gradient is disrupted, the kidney loses its ability to concentrate urine, resulting in greater water loss than sodium loss (18). If patient water intake is inadequate (due to diminished thirst sensation, confusion, or concomitant fluid restriction), hemoconcentration develops more readily, manifesting as hypernatremia. Following relief of obstruction, intrapelvic pressure rapidly decreases, glomerular filtration rate (eGFR) gradually recovers, concentrating function progressively restores, the medullary osmotic gradient is re-established and maintained, collecting duct water reabsorption capacity under ADH influence recovers, and serum sodium concentration gradually normalizes (2).

Animal studies have demonstrated that urinary tract obstruction interferes with sodium and water reabsorption through multiple mechanisms (19–21): (1) elevated intrapelvic pressure reduces medullary blood flow, disrupting the osmotic gradient; (2) downregulation of sodium transporters and aquaporin (AQP) expression; (3) local ischemia and inflammatory mediators (e.g., PGE_2_) inhibit sodium reabsorption; and (4) although the contralateral kidney compensatorily increases sodium excretion, it cannot compensate for the reabsorption deficit. This ultimately results in impaired sodium reabsorption, defective urinary concentration, and water loss, leading to hypernatremia.

## Conclusion

This patient initially presented with diabetic HHS and developed persistent hypernatremia after glycemic stabilization. Given his history of benign prostatic hyperplasia and urinary frequency-related fluid restriction, combined with abdominal CT findings suggestive of partial urinary tract obstruction, serum sodium normalized following surgical relief of obstruction. For unexplained hypernatremia, particularly in middle-aged and elderly male patients with concurrent hyperosmolar state, chronic kidney disease, or voiding abnormalities, partial urinary tract obstruction should be considered. This case also has limitations, such as the absence of a standard water deprivation test or ADH/copeptin measurement to definitively exclude partial diabetes insipidus, although normalization of serum sodium following relief of obstruction suggests obstruction as the primary cause. Early recognition and multidisciplinary collaborative intervention can help restore renal function, correct electrolyte disturbances, and prevent progression to obstructive nephropathy and renal failure. Regular urinary system ultrasonography, electrolyte monitoring, and post-void residual volume assessment are recommended for middle-aged and elderly patients with benign prostatic hyperplasia.

## Data Availability

The raw data supporting the conclusions of this article will be made available by the authors, without undue reservation.
